# Supplementary material for: NuA4 and H2A.Z control environmental responses and autotrophic growth in Arabidopsis
Source: Nat Commun. 2022 Jan 12;13:277. doi: 10.1038/s41467-021-27882-5 (PMC8755797; doi:10.1038/s41467-021-27882-5)
Supplement: Supplementary file 1 — Supplementary Information [file 41467_2021_27882_MOESM1_ESM.docx]

**NuA4 and H2A.Z control environmental responses and autotrophic growth in *Arabidopsis***

Bieluszewski *et al.*

**Supplementary Information**

**Supplementary Figures**

**Supplementary Fig. 1.** DNA sequence analysis of the *Ateaf1-1* allele. **a**, Gene structure diagram of the *AtEAF1* loci. Two different sequences targeted by gRNAs from the same genetic construct (#2) are marked with red and purple. Due to almost identical sequence of the two *AtEAF1* genes, each gRNA targeted both genes resulting in the total of four cut sites on each chromosome. **b**, A representative sequencing result of a genomic PCR clone from a single homozygous plant aligned with a fragment of the reference genomic sequences of *AtEAF1A and AtEAF1B.* All sequence alignment images in Supplementary Figures 1 and 2 were generated with Geneious 10 (Biomatters).

** Supplementary Fig. 2.** DNA sequence analysis of the *Atepl1a-2*, *Atepl1b-2* and *-3* alleles. **a**, Gene structure diagram of the *AtEPL1A* (At1g16690) and *AtEPL1B* (At1g79020) loci. Two different sequences targeted by gRNAs from the same genetic construct are marked with red and purple (*Atepl1a-2* and *Atepl1b-2*) or orange and cyan (*Atepl1b-3*). **b**, A representative sequencing result of a genomic PCR clone from a single homozygous plant aligned with a fragment of the reference genomic sequence of *AtEPL1A*. The deletion starts in the exon II and ends in the intron IV (downstream from the second cut site), therefore, the protein product cannot be predicted based on the genomic sequence. The target sequences are framed and color-coded. Black arrow heads mark Cas9-gRNA cut sites. **c**, A representative sequencing result of cDNA clones from a single homozygous mutant plant aligned with a fragment of the reference sequence of the *AtEPL1A* CDS. cDNA sequencing suggests that all transcripts are spliced in the same way which results in early translation termination. **d**, A representative sequencing result of a genomic PCR clone from a single *Atepl1b-2* homozygous plant aligned with a fragment of the genomic sequence of *AtEPL1B*. The target sequences are framed and color-coded. Black arrow heads mark Cas9-gRNA cut sites. **e**, A representative sequencing result of a genomic PCR clone from a single *Atepl1b-3* homozygous plant aligned with a fragment of the reference genomic sequence of *AtEPL1B*.

** Supplementary Fig. 3.** The phenotype of *Atepl1-1* is more severe than the *Atyaf9a-1 Atyaf9b-2* mutant phenotype. **a,** Quantitative description of 8-true leaf rosettes. Plants were grown on peat pellets (Jiffy) in the following conditions: 22°C, 16-hour light period, 40 μmol/(m^2^s) PAR, 70%. Graphs show statistics for petiole length (gray bars), blade length (green bars) and blade surface area (dark-green line) obtained from ten independent biological replicates for each value presented. Error bars represent standard deviation. Scale bar, 1cm. **b**, RT-qPCR analysis of relative gene expression in whole rosettes grown and collected as described above. Average expression levels from three independent replicates ± standard deviations are shown. Asterisks indicate significant differences relative to WT (two-tailed t-test, p < 0.05). **c**, Main stems and siliques of representative WT and *Atepl1* plants grown in the same conditions. Scale bars, 1 cm. Source data are provided as a Source Data file.

** Supplementary Fig. 4.** Photomorphogenesis in the *Atepl1-1* mutant. Apical curvature (**a**), hypocotyl length (**b**), cotyledon angle (**c**) and cotyledon surface (**d**) as measured 0, 6, 24 and 56 (for b and d) hours after dark-to-light transition. Boxplots represent statistics derived from at least five biological replicates. Each dot represents one individual. The center line indicates the median; upper and lower bounds indicate the 75th and 25th percentiles, respectively; and the whiskers indicate the minimum and maximum. *P* values were estimated by paired Welch *t* test. Source data are provided as a Source Data file.

** Supplementary Fig. 5.** The quadruple mutant *Atepl1-2 glk* (*Atepl1a-2 Atepl1b-2 glk1 glk2*) exhibits further reduction of expression of photosynthesis-related genes when compared to *Atepl1-2* (*Atepl1a-2 Atepl1b-2*) or *glk* (*glk1 glk2*) double mutants by RT-qPCR. Average expression levels from three independent replicates ± standard deviations are shown. Source data are provided as a Source Data file.

** Supplementary Fig. 6.** Early onset of the pathogen response-like transcriptomic pattern in *Atepl1-1*. **a**, Representative images of plant material collected for the RT-qPCR analysis. Approximately 30 seedlings were used for each of the three biological replicates. Scale bars, 1 cm. **b**, RT-qPCR analysis of relative gene expression in 2-true leaf seedlings (average expression levels from three independent replicates ± standard deviations) compared with RNA-seq data for 10-leaf rosettes obtained as described in Methods. Plants for RT-qPCR were grown on peat pellets (Jiffy) in the following conditions: 22°C, 16-hour light period, 40 μmol/(m^2^s) PAR, 70% RH. Source data are provided as a Source Data file.

** Supplementary Fig. 7.** Area proportional Venn diagrams illustrating overlaps between genes exhibiting the same direction of the expression change in *Atepl1-2*, *Ateaf1-1* and *glk1* mutants (blue and orange ovals) and the gene sets representing particular GO categories (gray ovals). Numeric values indicate numbers of genes present in a single sector of the diagram.

**Supplementary Fig. 8.** AtEPL1 supports nucleosomal H4 and H2A.Z acetylation in vitro. **a**, Purification of the recombinant *A. thaliana* picNuA4 from bacteria (Coomassie staining). Three different bacterial strains with polycistronic constructs carrying *At* picNuA4 components were used: 1) At+-His (His-HAM1, AtEPL1A-Flag, AtING2, AtEAF6) for initial tests, 2) At+-Flag (His-AtING2, Flag-HAM1, AtEAF6, AtEPL1A) for HAT assays, and 3) At- -Flag (with His-AtING2, Flag-HAM1, AtEAF6) for HAT assays without AtEPL1A. The first lane shows single step purification, while three other lanes show tandem purification. Numbers in brackets indicate expected molecular weights of the recombinant picNuA4 subunits. **b**, Comparison of HAT activity (counts per minute, CPM) as measured for SON and CHs. Error bars represent the range from two independent replicates. **c**, Western Blot test of αH2A.Zac (K4/K7/K11) antibody used in ChIP assays. Left panel. Human short oligonucleotides (SONs) enriched for HTA9 (Arabidopsis H2A.Z). SONs without HTA9 were used as a negative control. The Ab efficiently recognizes both human H2A.Zac and Arabidopsis H2A.Zac (HTA9ac). Bottom panel. αH4 Western Blot on the same membrane. Right panel. αFlag Western Blot on a twin membrane showing HTA9-Flag. Source data are provided as a Source Data file.

** Supplementary Fig. 9.** Alignment of H2A.Z histone protein sequences from different species. Only one representative protein was chosen for the alignment except *A. thaliana*, where all three functional copies were presented (HTA8, HTA9 and HTA11). Acetylation sites for human H2A.Z (HsH2A.Z) and budding yeast H2A.Z (ScH2A.Z) is indicated with cyan highlight according to ﻿Ishibashi et al. (2009) and Millar et al. (2006), respectively. Note the overall similarity for the histone core and significant differences for the N-tail, which is subjected to acetylation. A bootstrapped phylogenetic tree of the sequences used in the alignment is shown in the lower panel. BnH2A.Z, OsH2A.Z and HvH2A.Z stands for *Brassica napus*, *Oryza sativa* and *Hordeum vulgare* H2A.Z respectively.

** Supplementary Fig. 10.** ChIP-qPCR validation of the effect of *Atepl1-2* mutation on H4K5ac, H4K8ac, H4K12ac, H4K16ac, H2A.Zac and HTA9 (major H2A.Z protein) levels at seven loci: three downregulated (*RPS1*, *RPL11* and *RPL18*), one upregulated (At5g15710) and one unchanged (At5g16410) *in Atepl1-2*, and a gypsy element (At4g07700). Average expression levels from three independent replicates ± standard deviations are shown. All the qPCR measurements were normalized to H3 levels. Source data are provided as a Source Data file.

** Supplementary Fig. 11.** The changes in histone acetylation and total H2A.Z levels are different for genes misregulated in *Atepl1-2*. Comparison of spike-in-normalized H3K9ac, H4K5ac, H2A.Zac and total H2A.Z profiles between WT (blue) and *Atepl1-2* mutant (red). Particular plots show the profiles for all expressed (n=18620), upregulated in *Atepl1-2* (n=1746) and downregulated in *Atepl1-2* (n=975). Vertical axes values are relative. Note the different scale of changes in histone acetylation. Horizontal axes values represent chromosomal position relative to TSS.

** Supplementary Fig. 12.** Scatter plots showing correlations between chromatin marks (H3, H3K9ac, H4K5ac, H2A.Zac, total H2A.Z and unacetylated H2A.Z occupancy) and gene expression change (log_2_ fold change) as calculated by percentiles of expression change. Genes dysregulated in *Atepl1-2* (n=5135) were sorted based on their log_2_ fold change and divided into percentiles. For each percentile, the mean change in expression and the mean log_2_-transformed spike-in normalized ChIP-seq signal were calculated and plotted. To estimate values for non-acetylated H2A.Z, the ChIP-seq signal for acetylated H2A.Z were subtracted from the signal obtained for total H2A.Z.

** Supplementary Fig. 13.** Genes encoding plastid ribosomal proteins are significantly overrepresented among potentially NuA4-dependent genes. **a**, Ideogram of the NuA4-dependency determination process. **b**, Functional enrichment and relative expression of NuA4-dependent genes. *P* value for functional enrichment (column 5) is equivalent to EASE score (modified Fisher Exact test, see Methods). *P* values for the differences between median fold change against all genes downregulated in *Atepl1-1* (column 8) are results of Mann Whitney U test. GT stands for GO Term, UP stands for UniProt and KEGG stands for Kyoto Encyclopedia of Genes and Genomes.

**Supplementary Fig. 14.** Histone acetylation and H2A.Z profiles at TSS for selected gene groups upregulated in *Atepl1-2*. **a**, NuA4-dependent genes from each GO category (orange) were shown together with all the genes for this GO category (blue). Profiles in both WT and *Atepl1-2* background were shown next to each other. **b**, Profiles for NuA4-dependent genes (n= 347; orange) and all expressed genes (n= 18,620; blue) or downregulated in *Atepl1-2* (n= 941; red) were shown for comparison.

** Supplementary Fig. 15.** Upregulation of genes in NuA4 mutants is the consequence of both H2A.Z loss from gene bodies and increased levels of H3K9ac. **a** Boxplots of averaged H2A.Z enrichment in gene bodies for all genes expressed in WT (n= 18,620; blue) as well as genes downregulated (n= 941; red) and upregulated (n= 1746; green) in *Atepl1-2*. **b** Boxplots of averaged H2A.Z enrichment in gene bodies for all genes expressed in WT (n= 18,620; blue), and three subsections of genes upregulated in both *Atepl1-2* and *pie1* (n= 591), exclusively in *Atepl1-2* (n= 1836) and exclusively in *pie1* (n= 1705). **c** Spike-in-normalized H2A.Z, H2A.Zac and H3K9ac profiles across gene body for genes upregulated in both *pie1* and *Atepl1-2* (red), genes upregulated only in *Atepl1-2* (green), and genes upregulated only in *pie1* (blue) as measured by ChIP in WT. Corresponding profiles for all genes expressed in WT were shown as a reference (dashed line). **d** as for (c), but measured by ChIP in *Atepl1-2* mutant. For (a) and (b), center line indicates the median, upper and lower bounds represent the 75th and the 25th percentile, respectively, whiskers indicate the minimum and the maximum. Unpaired two- samples Wilcoxon test was used to determine significance.

**Supplementary Tables**

**Supplementary Table 1.** Median chloroplast size (corresponding to Figure 2A).

| **Median chloroplast projection surface area in µm^2^ (n chloroplasts / replicate = 20)** | | | | | | | |
| --- | --- | --- | --- | --- | --- | --- | --- |
| Repl. # | WT | *Atepl1-1* | *Atepl1-2* | *Atepl1-3* | *Ateaf1-1* | *glk* | *Atepl1 glk* |
| 1 | 37.11 | 11.19 | 11.02 | 21.09 | 19.26 | 11.22 | 7.95 |
| 2 | 39.59 | 23.42 | 23.77 | 18.94 | 26.48 | 16.32 | 6.10 |
| 3 | 28.51 | 11.25 | 12.22 | 15.44 | 27.75 | 14.32 | 7.63 |
| 4 | 29.34 | 11.02 | 17.82 | 16.32 | 16.29 | 13.26 | 4.45 |
| 5 | 29.19 | 16.44 | 12.02 | 16.50 | 17.70 | 13.17 | 4.86 |
| 6 | 32.11 | 17.20 | 11.84 | 15.17 | 20.71 | 10.99 | 4.09 |
| 7 | 27.34 | 16.85 | 11.37 | 14.64 | 15.91 | 10.93 | 5.98 |
| 8 | 35.38 | 16.11 | 10.16 | 15.73 | 13.67 | 15.14 | 6.75 |
| ***p* values (two-tailed Mann Whitney U test)** | | | | | | | |
|  | WT | *Atepl1-1* | *Atepl1-2* | *Atepl1-3* | *Ateaf1-1* | *glk* | *Atepl1 glk* |
| WT |  | 0.000155 | 0.000155 | 0.000155 | 0.000311 | 0.000155 | 0.000155 |
| *Atepl1-1* |  |  | 0.645377 | 0.798446 | 0.130381 | 0.160528 | 0.000155 |
| *Atepl1-2* |  |  |  | 0.064957 | 0.020668 | 0.878477 | 0.000155 |
| *Atepl1-3* |  |  |  |  | 0.234499 | 0.002953 | 0.000155 |
| *Ateaf1-1* |  |  |  |  |  | 0.002953 | 0.000155 |
| *glk* |  |  |  |  |  |  | 0.000155 |
| *Atepl1 glk* |  |  |  |  |  |  |  |

**Supplementary Table 2.** Median cell size (corresponding to Figure 2A).

| **Median cell projection surface area in µm^2^ (n cells / replicate = 9)** | | | | | | | |
| --- | --- | --- | --- | --- | --- | --- | --- |
| Repl. # | WT | *Atepl1-1* | *Atepl1-2* | *Atepl1-3* | *Ateaf1-1* | *glk* | *Atepl1 glk* |
| 1 | 3440.42 | 1357.28 | 1613.19 | 1610.72 | 1973.97 | 1796.00 | 979.53 |
| 2 | 3000.64 | 1856.62 | 1838.65 | 1248.41 | 2209.27 | 2395.61 | 612.16 |
| 3 | 1762.71 | 1168.82 | 1628.22 | 1271.33 | 2482.68 | 1904.75 | 759.08 |
| 4 | 2426.83 | 1157.92 | 1454.84 | 1423.20 | 1635.88 | 2228.77 | 2035.42 |
| 5 | 2658.77 | 1621.97 | 1040.92 | 1486.71 | 1791.99 | 1925.55 | 979.53 |
| 6 | 2528.16 | 1206.99 | 1370.12 | 1225.49 | 1661.38 | 1988.23 | 612.16 |
| 7 | 2226.82 | 1293.18 | 1558.29 | 1018.95 | 1539.85 | 2343.94 | 759.08 |
| 8 | 2284.62 | 1521.23 | 1299.72 | 1192.50 | 1691.55 | 2035.42 | 759.08 |
| ***p* values (two-tailed Mann Whitney U test)** | | | | | | | |
|  | WT | *Atepl1-1* | *Atepl1-2* | *Atepl1-3* | *Ateaf1-1* | *glk* | *Atepl1 glk* |
| WT |  | 0.000311 | 0.000311 | 0.000155 | 0.006993 | 0.049883 | 0.000311 |
| *Atepl1-1* |  |  | 0.441803 | 0.645377 | 0.004662 | 0.000311 | 0.010412 |
| *Atepl1-2* |  |  |  | 0.104895 | 0.010412 | 0.000311 | 0.010412 |
| *Atepl1-3* |  |  |  |  | 0.000311 | 0.000155 | 0.010412 |
| *Ateaf1-1* |  |  |  |  |  | 0.104895 | 0.004662 |
| *glk* |  |  |  |  |  |  | 0.001865 |
| *Atepl1 glk* |  |  |  |  |  |  |  |

**Supplementary Table 3.** Total chlorophyll (corresponding to Figure 2B).

| **Total Chlorophyll (nmol total chlorophyll / mg fresh weight)** | | | | | | | |
| --- | --- | --- | --- | --- | --- | --- | --- |
|  | WT | *Atepl1-1* | *Atepl1-2* | *Atepl1-3* | *Ateaf1-1* | *glk* | *Atepl1 glk* |
|  | 1.36 | 0.58 | 0.55 | 0.60 | 0.62 | 0.45 | 0.30 |
|  | 1.29 | 0.52 | 0.54 | 0.61 | 0.60 | 0.39 | 0.33 |
|  | 1.51 | 0.58 | 0.48 | 0.60 | 0.61 | 0.52 | 0.31 |
| Mean | 1.39 | 0.56 | 0.53 | 0.61 | 0.61 | 0.45 | 0.31 |
| alpha | 0.05 | 0.05 | 0.05 | 0.05 | 0.05 | 0.05 | 0.05 |
| SD | 0.11 | 0.03 | 0.04 | 0.01 | 0.01 | 0.07 | 0.02 |
| n | 3 | 3 | 3 | 3 | 3 | 3 | 3 |
| CI 95% | 0.28 | 0.08 | 0.10 | 0.02 | 0.03 | 0.16 | 0.04 |
| ***p* values (two-tailed T-test)** | | | | | | | |
|  | WT | *Atepl1-1* | *Atepl1-2* | *Atepl1-3* | *Ateaf1-1* | *glk* | *Atepl1 glk* |
| WT |  | 0.00026 | 0.00023 | 0.00028 | 0.00029 | 0.00024 | 0.00008 |
| *Atepl1-1* |  |  | 0.29529 | 0.09824 | 0.07235 | 0.06581 | 0.00030 |
| *Atepl1-2* |  |  |  | 0.02606 | 0.02122 | 0.18212 | 0.00086 |
| *Atepl1-3* |  |  |  |  | 0.41410 | 0.01673 | 0.00001 |
| *Ateaf1-1* |  |  |  |  |  | 0.01478 | 0.00001 |
| *glk* |  |  |  |  |  |  | 0.01964 |
| *Atepl1 glk* |  |  |  |  |  |  |  |

**Supplementary Table 4.** Chlorophyll a / b ratio (corresponding to Figure 2B).

| **Chlorophyll a / b ratio** | | | | | | | |
| --- | --- | --- | --- | --- | --- | --- | --- |
|  | WT | *Atepl1-1* | *Atepl1-2* | *Atepl1-3* | *Ateaf1-1* | *glk* | *Atepl1 glk* |
|  | 3.34 | 3.21 | 2.91 | 3.44 | 3.22 | 4.92 | 3.67 |
|  | 3.31 | 3.12 | 3.27 | 2.98 | 3.22 | 4.66 | 3.76 |
|  | 3.26 | 3.07 | 2.96 | 3.01 | 3.32 | 4.85 | 3.49 |
| Mean | 3.30 | 3.13 | 3.05 | 3.14 | 3.25 | 4.81 | 3.64 |
| alpha | 0.05 | 0.05 | 0.05 | 0.05 | 0.05 | 0.05 | 0.05 |
| SD | 0.04 | 0.07 | 0.19 | 0.26 | 0.06 | 0.14 | 0.14 |
| n | 3 | 3 | 3 | 3 | 3 | 3 | 3 |
| CI 95% | 0.10 | 0.19 | 0.48 | 0.64 | 0.14 | 0.34 | 0.35 |
| ***p* values (two-tailed T-test)** | | | | | | | |
|  | WT | *Atepl1-1* | *Atepl1-2* | *Atepl1-3* | *Ateaf1-1* | *glk* | *Atepl1 glk* |
| WT |  | 0.02508 | 0.08391 | 0.34148 | 0.27991 | 0.00005 | 0.01634 |
| *Atepl1-1* |  |  | 0.49917 | 0.95560 | 0.09151 | 0.00005 | 0.00525 |
| *Atepl1-2* |  |  |  | 0.62631 | 0.14550 | 0.00020 | 0.01239 |
| *Atepl1-3* |  |  |  |  | 0.50408 | 0.00057 | 0.04253 |
| *Ateaf1-1* |  |  |  |  |  | 0.00005 | 0.01160 |
| *glk* |  |  |  |  |  |  | 0.00048 |
| *Atepl1 glk* |  |  |  |  |  |  |  |

**Supplementary Table 5.** Rosette size in week 2 after germination (corresponding to Figure 2C).

| **Log_10_ of rosette radius in mm** | | | | | | | |
| --- | --- | --- | --- | --- | --- | --- | --- |
| **Week 2** | | | | | | | |
| Plant # | WT | *Atepl1-1* | *Atepl1-2* | *Atepl1-3* | *Ateaf1-1* | *glk* | *Atepl1 glk* |
| 1 | 0.89 | 0.18 | 0.20 | 0.38 | 0.44 | 0.77 | 0.36 |
| 2 | 0.87 | 0.10 | 0.16 | 0.31 | 0.47 | 0.73 | 0.20 |
| 3 | 0.85 | 0.10 | 0.35 | 0.21 | 0.39 | 0.80 | 0.33 |
| 4 | 1.01 | 0.41 | 0.37 | -0.05 | 0.19 | 0.72 | 0.34 |
| 5 | 0.78 | 0.36 | 0.33 | 0.35 | 0.42 | 0.74 | 0.14 |
| 6 | 0.64 | 0.28 | 0.11 | 0.31 | 0.40 | 0.72 | 0.25 |
| 7 | 0.78 | 0.39 | 0.36 | 0.28 | 0.40 | 0.73 | 0.12 |
| 8 | 0.76 | 0.29 | 0.25 | 0.40 | 0.29 | 0.68 | 0.02 |
| 9 | 0.83 | 0.38 | 0.19 | 0.20 | 0.32 | 0.70 | 0.23 |
| 10 | 0.87 | 0.13 | 0.15 | 0.28 | 0.33 | 0.57 | 0.16 |
| 11 |  |  | 0.20 |  |  |  |  |
| 12 |  |  | 0.17 |  |  |  |  |
| 13 |  |  | 0.33 |  |  |  |  |
| 14 |  |  | 0.33 |  |  |  |  |
| 15 |  |  | 0.21 |  |  |  |  |
| 16 |  |  | 0.27 |  |  |  |  |
| 17 |  |  | 0.36 |  |  |  |  |
| 18 |  |  | 0.37 |  |  |  |  |
| 19 |  |  | 0.34 |  |  |  |  |
| 20 |  |  | 0.22 |  |  |  |  |
| Mean | 0.83 | 0.26 | 0.26 | 0.27 | 0.37 | 0.72 | 0.22 |
| alpha | 0.05 | 0.05 | 0.05 | 0.05 | 0.05 | 0.05 | 0.05 |
| SD | 0.10 | 0.13 | 0.09 | 0.13 | 0.08 | 0.06 | 0.11 |
| n | 10 | 10 | 20 | 10 | 10 | 10 | 10 |
| CI 95% | 0.07 | 0.09 | 0.04 | 0.09 | 0.06 | 0.04 | 0.08 |
| ***p* values (two-tailed T-test)** | | | | | | | |
|  | WT | *Atepl1-1* | *Atepl1-2* | *Atepl1-3* | *Ateaf1-1* | *glk* | *Atepl1 glk* |
| WT |  | 1.32E-09 | 9.88E-16 | 2.29E-09 | 1.19E-09 | 7.05E-03 | 1.13E-10 |
| *Atepl1-1* |  |  | 9.55E-01 | 9.12E-01 | 4.21E-02 | 5.48E-09 | 3.98E-01 |
| *Atepl1-2* |  |  |  | 9.16E-01 | 4.64E-03 | 1.00E-14 | 2.03E-01 |
| *Atepl1-3* |  |  |  |  | 6.06E-02 | 1.08E-08 | 3.47E-01 |
| *Ateaf1-1* |  |  |  |  |  | 3.31E-09 | 3.05E-03 |
| *glk* |  |  |  |  |  |  | 2.50E-10 |
| *Atepl1 glk* |  |  |  |  |  |  |  |

**Supplementary Table 6.** Rosette size in week 3 after germination (corresponding to Figure 2C).

| **Log_10_ of rosette radius in mm** | | | | | | | |
| --- | --- | --- | --- | --- | --- | --- | --- |
| **Week 3** | | | | | | | |
| Plant # | WT | *Atepl1-1* | *Atepl1-2* | *Atepl1-3* | *Ateaf1-1* | *glk* | *Atepl1 glk* |
| 1 | 1.35 | 0.51 | 0.47 | 0.58 | 0.67 | 1.16 | 0.49 |
| 2 | 1.45 | 0.24 | 0.36 | 0.65 | 0.85 | 1.11 | 0.29 |
| 3 | 1.38 | 0.51 | 0.71 | 0.49 | 0.82 | 1.14 | 0.46 |
| 4 | 1.51 | 0.71 | 0.60 | 0.24 | 0.54 | 1.15 | 0.47 |
| 5 | 1.40 | 0.58 | 0.48 | 0.59 | 0.78 | 1.11 | 0.32 |
| 6 | 1.30 | 0.46 | 0.19 | 0.53 | 0.79 | 1.10 | 0.42 |
| 7 | 1.38 | 0.72 | 0.60 | 0.53 | 0.71 | 1.10 | 0.28 |
| 8 | 1.34 | 0.55 | 0.43 | 0.65 | 0.63 | 1.06 | 0.18 |
| 9 | 1.39 | 0.69 | 0.42 | 0.48 | 0.63 | 1.10 | 0.42 |
| 10 | 1.38 | 0.31 | 0.38 | 0.59 | 0.61 | 0.98 | 0.39 |
| 11 |  |  | 0.48 |  |  |  |  |
| 12 |  |  | 0.42 |  |  |  |  |
| 13 |  |  | 0.60 |  |  |  |  |
| 14 |  |  | 0.69 |  |  |  |  |
| 15 |  |  | 0.52 |  |  |  |  |
| 16 |  |  | 0.49 |  |  |  |  |
| 17 |  |  | 0.64 |  |  |  |  |
| 18 |  |  | 0.53 |  |  |  |  |
| 19 |  |  | 0.57 |  |  |  |  |
| 20 |  |  | 0.43 |  |  |  |  |
| Mean | 1.39 | 0.53 | 0.50 | 0.53 | 0.70 | 1.10 | 0.37 |
| alpha | 0.05 | 0.05 | 0.05 | 0.05 | 0.05 | 0.05 | 0.05 |
| SD | 0.06 | 0.16 | 0.12 | 0.12 | 0.10 | 0.05 | 0.10 |
| n | 10 | 10 | 20 | 10 | 10 | 10 | 10 |
| CI 95% | 0.04 | 0.11 | 0.06 | 0.09 | 0.07 | 0.04 | 0.07 |
| ***p* values (two-tailed T-test)** | | | | | | | |
|  | WT | *Atepl1-1* | *Atepl1-2* | *Atepl1-3* | *Ateaf1-1* | *glk* | *Atepl1 glk* |
| WT |  | 4.46E-12 | 6.08E-19 | 6.20E-14 | 3.48E-13 | 6.89E-10 | 2.54E-16 |
| *Atepl1-1* |  |  | 5.76E-01 | 9.64E-01 | 9.95E-03 | 2.91E-09 | 1.66E-02 |
| *Atepl1-2* |  |  |  | 4.93E-01 | 1.16E-04 | 1.12E-14 | 8.54E-03 |
| *Atepl1-3* |  |  |  |  | 2.99E-03 | 4.79E-11 | 4.26E-03 |
| *Ateaf1-1* |  |  |  |  |  | 1.96E-09 | 8.22E-07 |
| *glk* |  |  |  |  |  |  | 5.80E-14 |
| *Atepl1 glk* |  |  |  |  |  |  |  |

**Supplementary Table 7.** Rosette size in week 4 after germination (corresponding to Figure 2C).

| **Log10 of rosette radius in mm** | | | | | | | |
| --- | --- | --- | --- | --- | --- | --- | --- |
| **Week 4** | | | | | | | |
| Plant # | WT | *Atepl1-1* | *Atepl1-2* | *Atepl1-3* | *Ateaf1-1* | *glk* | *Atepl1 glk* |
| 1 | 1.69 | 0.87 | 0.82 | 0.88 | 1.00 | 1.48 | 0.73 |
| 2 | 1.75 | 0.69 | 0.52 | 0.86 | 1.06 | 1.39 | 0.56 |
| 3 | 1.71 | 0.82 | 0.82 | 0.72 | 1.06 | 1.49 | 0.69 |
| 4 | 1.74 | 0.96 | 0.82 | 0.65 | 0.88 | 1.49 | 0.69 |
| 5 | 1.70 | 0.88 | 0.83 | 0.86 | 1.02 | 1.45 | 0.68 |
| 6 | 1.69 | 0.89 | 0.28 | 0.82 | 1.04 | 1.42 | 0.61 |
| 7 | 1.71 | 0.94 | 0.83 | 0.79 | 0.92 | 1.44 | 0.54 |
| 8 | 1.69 | 0.82 | 0.73 | 0.93 | 0.90 | 1.42 | 0.28 |
| 9 | 1.67 | 0.94 | 0.76 | 0.85 | 0.93 | 1.42 | 0.60 |
| 10 | 1.68 | 0.62 | 0.77 | 0.89 | 0.84 | 1.35 | 0.69 |
| 11 |  |  | 0.86 |  |  |  |  |
| 12 |  |  | 0.69 |  |  |  |  |
| 13 |  |  | 0.87 |  |  |  |  |
| 14 |  |  | 0.87 |  |  |  |  |
| 15 |  |  | 0.80 |  |  |  |  |
| 16 |  |  | 0.84 |  |  |  |  |
| 17 |  |  | 0.86 |  |  |  |  |
| 18 |  |  | 0.83 |  |  |  |  |
| 19 |  |  | 0.93 |  |  |  |  |
| 20 |  |  | 0.70 |  |  |  |  |
| Mean | 1.70 | 0.84 | 0.77 | 0.83 | 0.96 | 1.43 | 0.61 |
| alpha | 0.05 | 0.05 | 0.05 | 0.05 | 0.05 | 0.05 | 0.05 |
| SD | 0.03 | 0.11 | 0.15 | 0.09 | 0.08 | 0.04 | 0.13 |
| n | 10 | 10 | 20 | 10 | 10 | 10 | 10 |
| CI 95% | 0.02 | 0.08 | 0.07 | 0.06 | 0.06 | 0.03 | 0.09 |
|  | | | | | | | |
|  | WT | *Atepl1-1* | *Atepl1-2* | *Atepl1-3* | *Ateaf1-1* | *glk* | *Atepl1 glk* |
| WT |  | 5.33E-15 | 4.80E-18 | 4.32E-17 | 3.77E-16 | 3.23E-12 | 9.90E-16 |
| *Atepl1-1* |  |  | 1.82E-01 | 6.78E-01 | 1.26E-02 | 7.56E-12 | 4.01E-04 |
| *Atepl1-2* |  |  |  | 2.97E-01 | 5.85E-04 | 4.03E-14 | 5.73E-03 |
| *Atepl1-3* |  |  |  |  | 1.44E-03 | 9.84E-14 | 3.49E-04 |
| *Ateaf1-1* |  |  |  |  |  | 4.03E-12 | 8.08E-07 |
| *glk* |  |  |  |  |  |  | 2.47E-13 |
| *Atepl1 glk* |  |  |  |  |  |  |  |

**Supplementary Table 8.** Attempts to obtain triple mutants *Atepl1a Atepl1b arp6* and *Atepl1a Atepl1b mbd9*. All the genes are not genetically linked.

| F_1_ cross | No. of plants genotyped in F_2_ | No. of triple mutant expected | No. of triple mutants found | Genotype selected for next generation | No. of plants genotyped in F_3_ | No. of triple mutants expected | No. of triple mutant plants |
| --- | --- | --- | --- | --- | --- | --- | --- |
| *Atepl1-1* × *arp6* | 285 | 4 (1/64) | 0 | *Atepl1a*^-/-^ *Atepl1*^+/-^ *arp6*^-/-^ | 219 | 55 (1/4) | 0 |
| *Atepl1-2* × *mbd9-1* | 267 | 4 (1/64) | 0 | *Atepl1a*^-/-^ *Atepl1*^+/-^ *mbd9*^-/-^ | 125 | 31 (1/4) | 0 |

**Supplementary Table 9.** Functional enrichment and relative expression of NuA4-dependent genes.

*P* value for functional enrichment (column 5) is equivalent to EASE score (modified Fisher Exact test, see Methods). *P* values for the differences between median fold change against all genes downregulated in *Atepl1-2* (column 8) are results of Mann Whitney U test. GT stands for GO Term, UP stands for UniProt and KEGG stands for Kyoto Encyclopedia of Genes and Genomes.

**Supplementary Table 10.** ChIP-seq summary.

| **Sample** | **Antibody** | **Total**  **reads** | **Mapped**  **reads** | **Duplicates** | **Analyzed**  **reads** | **Mouse**  **reads** | **Fraction of mouse** | **Spike-in** |
| --- | --- | --- | --- | --- | --- | --- | --- | --- |
| epl2_H3 | H3 | 21368578 | 19748913 | 5495889 | 14253024 | 537809 | 0.0364 | 0.0324 |
| epl3_H3 | H3 | 19300672 | 17869582 | 5371442 | 12498140 | 399292 | 0.0310 |  |
| epl4_H3 | H3 | 20967261 | 18446233 | 6138110 | 12308123 | 380419 | 0.0300 |  |
| WT2_1_H3 | H3 | 22194295 | 21392573 | 5947892 | 15444681 | 522769 | 0.0327 | 0.0373 |
| WT3_H3 | H3 | 20235352 | 19536076 | 6273084 | 13262992 | 502727 | 0.0365 |  |
| WT4_H3 | H3 | 22392052 | 21504402 | 6620439 | 14883963 | 660968 | 0.0425 |  |
| epl2_H3K9ac | H3K9ac | 6482922 | 5300353 | 1456456 | 3843897 | 127218 | 0.0320 | Not used |
| epl3_H3K9ac | H3K9ac | 17265676 | 13782719 | 4575676 | 9207043 | 145810 | 0.0156 | 0.0172 |
| epl4_H3K9ac | H3K9ac | 19219574 | 13684182 | 3461175 | 10223007 | 195414 | 0.0188 |  |
| WT2_1_H3K9ac | H3K9ac | 18074380 | 16849044 | 4838713 | 12010331 | 219056 | 0.0179 | 0.0185 |
| WT3_H3K9ac | H3K9ac | 18639713 | 16844507 | 5677908 | 11166599 | 171393 | 0.0151 |  |
| WT4_H3K9ac | H3K9ac | 18395451 | 16431382 | 5497196 | 10934186 | 251640 | 0.0225 |  |
| epl2_H4K5ac | H4K5ac | 19861888 | 10439005 | 3263945 | 7175060 | 1089966 | 0.1319 | 0.1338 |
| epl3_H4K5ac | H4K5ac | 19481167 | 10547446 | 2839972 | 7707474 | 787961 | 0.0928 |  |
| epl4_H4K5ac | H4K5ac | 21824904 | 8028253 | 2410606 | 5617647 | 1206501 | 0.1768 |  |
| WT2_1_H4K5ac | H4K5ac | 18713901 | 16778202 | 4544722 | 12233480 | 454494 | 0.0358 | 0.0634 |
| WT2_H4K5ac | H4K5ac | 17315277 | 15632424 | 4949223 | 10683201 | 643418 | 0.0568 |  |
| WT4_H4K5ac | H4K5ac | 17765646 | 15169934 | 4889503 | 10280431 | 1110910 | 0.0975 |  |
| epl2_H2A.Zac | H2A.Zac | 23442785 | 4210622 | 3348883 | 861739 | 1627452 | 0.653808 | 0.6444 |
| epl3_H2A.Zac | H2A.Zac | 20899021 | 4269884 | 3065361 | 1204523 | 2094783 | 0.634916 |  |
| WT1_ H2A.Zac | H2A.Zac | 15597180 | 11274719 | 1758956 | 9515763 | 3494955 | 0.268621 | 0.3994 |
| WT3_ H2A.Zac | H2A.Zac | 13790395 | 6363852 | 1326357 | 5037495 | 5688685 | 0.530355 |  |
| WT4_ H2A.Zac | H2A.Zac | 17362714 | 1619213 | 886697 | 732516 | 1289254 | 0.637686 | Not used |
| EPL2_H2A.Z-T | HTA9 | 21746989 | 5669264 | 3600109 | 2069155 | 255912 | 0.110067 | 0.1056 |
| EPL3_ H2A.Z-T | HTA9 | 19919782 | 2575248 | 1853007 | 722241 | 175057 | 0.195093 | Not used |
| EPL4_ H2A.Z-T | HTA9 | 17405211 | 7282787 | 5244441 | 2038346 | 229178 | 0.10107 |  |
| WT1_ H2A.Z-T | HTA9 | 13936733 | 12863439 | 1496831 | 11366608 | 803313 | 0.066008 | 0.0638 |
| WT3_ H2A.Z-T | HTA9 | 20750557 | 19195245 | 2917210 | 16278035 | 1068660 | 0.061606 |  |
| WT5_ H2A.Z-T | HTA9 | 21465393 | 4707713 | 2290967 | 2416746 | 500611 | 0.171597 | Not used |
