# Supplementary material for: NuA4 and H2A.Z control environmental responses and autotrophic growth in Arabidopsis
Source: Nat Commun. 2022 Jan 12;13:277. doi: 10.1038/s41467-021-27882-5 (PMC8755797; doi:10.1038/s41467-021-27882-5)
Supplement: Supplementary file 3 — Description of Additional Supplementary Files [file 41467_2021_27882_MOESM3_ESM.pdf]

Description of Additional Supplementary Files

File Name: Supplementary Data 1
Description: All nuclear genes showing at least 1.5- fold reduction in transcript level in Atepl1-2 as determined by RNA-seq

File Name: Supplementary Data 2
Description: All nuclear genes showing at least 1.5- fold reduction in transcript level in glk as determined by RNAseq

File Name: Supplementary Data 3
Description: All nuclear genes showing at least 1.5- fold reduction in transcript level in Ateaf1-1 as determined by RNA-seq

File Name: Supplementary Data 4
Description: All nuclear genes showing at least 1.5- fold increase in transcript level in Atepl1-2 as determined by RNA-seq

File Name: Supplementary Data 5
Description: All nuclear genes showing at least 1.5- fold increase in transcript level in glk as determined by RNAseq

File Name: Supplementary Data 6
Description: All nuclear genes showing at least 1.5- fold increase in transcript level in Ateaf1-1 as determined by RNA-seq

File Name: Supplementary Data 7
Description: Genes downregulated in glk, Atepl1-2 or Ateaf1-1 mapped to GO IDs.

File Name: Supplementary Data 8
Description: PANTHER Overrepresentation Test of genes downregulated in Atepl1-2 against GO biological process

File Name: Supplementary Data 9
Description: PANTHER Overrepresentation Test of genes downregulated in Atepl1-2 against GO cellular component

File Name: Supplementary Data 10
Description: Genes upregulated in glk, Atepl1-2 or Ateaf1-1 mapped to GO IDs.

File Name: Supplementary Data 11
Description: PANTHER Overrepresentation Test of genes upregulated in Atepl1-2 against GO biological process

File Name: Supplementary Data 12
Description: PANTHER Overrepresentation Test of genes upregulated in Atepl1-2 against GO cellular component

File Name: Supplementary Data 13
Description: List of significantly identified proteins in AtEPL1B TAP #1 samples.

File Name: Supplementary Data 14
Description: List of significantly identified proteins in AtEPL1B TAP #2 samples.

File Name: Supplementary Data 15
Description: Protein identification details for AtEPL1B TAP #1 samples.

File Name: Supplementary Data 16
Description: Protein identification details for AtEPL1B TAP #2 samples.

File Name: Supplementary Data 17
Description: Selection of NuA4- dependent genes based on changes in transcription and acetylation of H3K9 and H4K5

File Name: Supplementary Data 18
Description: GO analysis of NuA4 dependent genes carried out with use of DAVID annotation tool

File Name: Supplementary Data 19
Description: List of oligonucleotides used in the manuscript
