# Supplementary material for: NuA4 and H2A.Z control environmental responses and autotrophic growth in Arabidopsis
Source: Nat Commun. 2022 Jan 12;13:277. doi: 10.1038/s41467-021-27882-5 (PMC8755797; doi:10.1038/s41467-021-27882-5)
Supplement: Supplementary file 23 — Reporting Summary [file 41467_2021_27882_MOESM23_ESM.pdf]

nature research

Corresponding author(s): Piotr A. Ziolkowski, Jacques Côté

Last updated by author(s): Dec 20, 2021

Reporting Summary

Nature Research wishes to improve the reproducibility of the work that we publish. This form provides structure for consistency and transparency in reporting. For further information on Nature Research policies, see Authors & Referees and the Editorial Policy Checklist .

Statistics

For all statistical analyses, confirm that the following items are present in the figure legend, table legend, main text, or Methods section.

n/a	Confirmed	
☐	☒	The exact sample size (*n*) for each experimental group/condition, given as a discrete number and unit of measurement
☐	☒	A statement on whether measurements were taken from distinct samples or whether the same sample was measured repeatedly
☐	☒	The statistical test(s) used AND whether they are one- or two-sided<br>*Only common tests should be described solely by name; describe more complex techniques in the Methods section.*
☐	☒	A description of all covariates tested
☒	☐	A description of any assumptions or corrections, such as tests of normality and adjustment for multiple comparisons
☐	☒	A full description of the statistical parameters including central tendency (e.g. means) or other basic estimates (e.g. regression coefficient) AND variation (e.g. standard deviation) or associated estimates of uncertainty (e.g. confidence intervals)
☐	☒	For null hypothesis testing, the test statistic (e.g. *F*, *t*, *r*) with confidence intervals, effect sizes, degrees of freedom and *P* value noted<br>*Give P values as exact values whenever suitable.*
☒	☐	For Bayesian analysis, information on the choice of priors and Markov chain Monte Carlo settings
☒	☐	For hierarchical and complex designs, identification of the appropriate level for tests and full reporting of outcomes
☒	☐	Estimates of effect sizes (e.g. Cohen's *d*, Pearson's *r*), indicating how they were calculated

Our web collection on statistics for biologists contains articles on many of the points above.

Software and code

Policy information about availability of computer code

Data collection	All data was managed with Excel (Microsoft Office 365),R 3.6.2 (www.R-project.org) and RStudio 1.4.1717 (RStudio).
Data analysis	All calculations and statistical tests not described in RNA-seq or ChIP-seq sections were run and plotted in Excel (Microsoft Office 365), except Mann-Whitney U tests run with an online calculator (http://www.statskingdom.com). For analysis of RNA-seq analysis, BBduk2 (https://jgi.doe.gov/data-and-tools/bbtools), Salmon 1.3 and limma R package were used. Gene Ontology analysis was carried out using Panther (http://pantherdb.org) and DAVID (https://david.ncifcrf.gov). For analysis of ChIP-seq, BBduk2 , bwa 0.7.17, Sambamba 0.7.0, BamTools 2.5.1 and DANPOS 2.2.1 (Kaifu Chen, Baylor College of Medicine) were used. Plots presented in Figures 2 A were created with BoxPlotR (http://shiny.chemgrid.org/boxplotr/). Plots presented in Figure 2B, Figure 5, Figure 6, Figure 7A,D,E,F, Figure 8A,B,D,E, Supplementary Figures 3-6, and Supplementary Figures 10-15 were created using R 3.6.2 (www.R-project.org) and RStudio 1.4.1717 (RStudio). Figure 2C, 3A, 3C were plotted in Excel (Microsoft Office 365). For creation of the area proportional Venn diagrams presented in Figure 3 B and Supplementary Figure 7 the gene sets included in Supplementary Files were first analyzed for overlaps by BioVenn (https://www.biovenn.nl/index.php), then plotted with EulerAPE 2.0.3 (https://www.eulerdiagrams.org/eulerAPE/) and finally graphically adjusted (coloring, line formatting) in PowerPoint (Microsoft Office 365). Raw Sanger sequencing DNA reads were analyzed with Geneious 10.2.6 (Biomatters Ltd.).

For manuscripts utilizing custom algorithms or software that are central to the research but not yet described in published literature, software must be made available to editors/reviewers. We strongly encourage code deposition in a community repository (e.g. GitHub). See the Nature Research guidelines for submitting code & software for further information.

Data

Policy information about availability of data

All manuscripts must include a data availability statement. This statement should provide the following information, where applicable:

- Accession codes, unique identifiers, or web links for publicly available datasets
- A list of figures that have associated raw data
- A description of any restrictions on data availability

Gene lists used for preparation of Figures 3, Supplementary Figures 7 and 14 were downloaded from TAIR (www.arabidopsis.org) with Gene Functional Annotation tool. Sequences of HTA8, HTA9 and HTA9 shown in Supplementary Figure 9 of Arabidopsis were downloaded from TAIR (www.arabidopsis.org). Gene sequences of H2A.Z in B. napus, H.vulgare and O. sativa presented in Supplementary Figure 9, were downloaded from NCBI (www.ncbi.nlm.nih.gov). List of upregulated genes in Arabidopsis pie1-5 mutant presented on Figure 8C, was obtained from "SWR1 Chromatin-Remodeling Complex Subunits and H2A.Z Have Non-overlapping Functions in Immunity and Gene Regulation in Arabidopsis" by S. Berriri et al., 2016. Raw sequencing reads from RNA-Seq and ChIP-seq were deposited into GEO database and are publicly available via this link: https://www.ncbi.nlm.nih.gov/geo/query/acc.cgi?acc=GSE152940. AP-MS/MS data have been deposited in Proteomics Identifications Database (PRIDE) under accession number PXD030302 and are publicly available via this link: https://www.ebi.ac.uk/pride/archive/projects/PXD030302. All data generated or analysed during this study are included in this published article (and its Supplementary Information and Source Data file) except for raw data related to confocal microscopy (available from the corresponding author on reasonable request), AP-MS/MS, RNA-seq and ChIP-seq data, deposited in public databases (referenced above).

Field-specific reporting

Please select the one below that is the best fit for your research. If you are not sure, read the appropriate sections before making your selection.

- [x] Life sciences
- [ ] Behavioural & social sciences
- [ ] Ecological, evolutionary & environmental sciences

For a reference copy of the document with all sections, see nature.com/documents/nr-reporting-summary-flat.pdf

Life sciences study design

All studies must disclose on these points even when the disclosure is negative.

Sample size	For all experiments sample sizes were determined in advance. Due to large number of genotypes compared in parallel, sample sizes were in all cases constrained by practical reasons. RNA-seq and ChIP-seq experiments were carried out in triplicates according to standard practice in the field.
Data exclusions	One biological replicate of ChIP-seq for H3K9ac and H2A.Z for Atepl1-2 was not used due to low reads number. One biological replicate of ChIP-seq for H2A.Z and H2A.Zac for wild-type was not used due to low reads number.
Replication	All experiments were repeated at least 3 times with consistent results.
Randomization	Not considered necessary and not used.
Blinding	Not considered necessary and not used.

Behavioural & social sciences study design

All studies must disclose on these points even when the disclosure is negative.

Study description	*Briefly describe the study type including whether data are quantitative, qualitative, or mixed-methods (e.g. qualitative cross-sectional, quantitative experimental, mixed-methods case study).*
Research sample	*State the research sample (e.g. Harvard university undergraduates, villagers in rural India) and provide relevant demographic information (e.g. age, sex) and indicate whether the sample is representative. Provide a rationale for the study sample chosen. For studies involving existing datasets, please describe the dataset and source.*
Sampling strategy	*Describe the sampling procedure (e.g. random, snowball, stratified, convenience). Describe the statistical methods that were used to predetermine sample size OR if no sample-size calculation was performed, describe how sample sizes were chosen and provide a rationale for why these sample sizes are sufficient. For qualitative data, please indicate whether data saturation was considered, and what criteria were used to decide that no further sampling was needed.*
Data collection	*Provide details about the data collection procedure, including the instruments or devices used to record the data (e.g. pen and paper, computer, eye tracker, video or audio equipment) whether anyone was present besides the participant(s) and the researcher, and whether the researcher was blind to experimental condition and/or the study hypothesis during data collection.*
Timing	*Indicate the start and stop dates of data collection. If there is a gap between collection periods, state the dates for each sample cohort.*

Data exclusions	*If no data were excluded from the analyses, state so OR if data were excluded, provide the exact number of exclusions and the rationale behind them, indicating whether exclusion criteria were pre-established.*
Non-participation	*State how many participants dropped out/declined participation and the reason(s) given OR provide response rate OR state that no participants dropped out/declined participation.*
Randomization	*If participants were not allocated into experimental groups, state so OR describe how participants were allocated to groups, and if allocation was not random, describe how covariates were controlled.*

Ecological, evolutionary & environmental sciences study design

All studies must disclose on these points even when the disclosure is negative.

Study description	*Briefly describe the study. For quantitative data include treatment factors and interactions, design structure (e.g. factorial, nested, hierarchical), nature and number of experimental units and replicates.*
Research sample	*Describe the research sample (e.g. a group of tagged Passer domesticus, all Stenocereus thurberi within Organ Pipe Cactus National Monument), and provide a rationale for the sample choice. When relevant, describe the organism taxa, source, sex, age range and any manipulations. State what population the sample is meant to represent when applicable. For studies involving existing datasets, describe the data and its source.*
Sampling strategy	*Note the sampling procedure. Describe the statistical methods that were used to predetermine sample size OR if no sample-size calculation was performed, describe how sample sizes were chosen and provide a rationale for why these sample sizes are sufficient.*
Data collection	*Describe the data collection procedure, including who recorded the data and how.*
Timing and spatial scale	*Indicate the start and stop dates of data collection, noting the frequency and periodicity of sampling and providing a rationale for these choices. If there is a gap between collection periods, state the dates for each sample cohort. Specify the spatial scale from which the data are taken*
Data exclusions	*If no data were excluded from the analyses, state so OR if data were excluded, describe the exclusions and the rationale behind them, indicating whether exclusion criteria were pre-established.*
Reproducibility	*Describe the measures taken to verify the reproducibility of experimental findings. For each experiment, note whether any attempts to repeat the experiment failed OR state that all attempts to repeat the experiment were successful.*
Randomization	*Describe how samples/organisms/participants were allocated into groups. If allocation was not random, describe how covariates were controlled. If this is not relevant to your study, explain why.*
Blinding	*Describe the extent of blinding used during data acquisition and analysis. If blinding was not possible, describe why OR explain why blinding was not relevant to your study.*

Did the study involve field work? ☐ Yes ☐ No

Field work, collection and transport

Field conditions	*Describe the study conditions for field work, providing relevant parameters (e.g. temperature, rainfall).*
Location	*State the location of the sampling or experiment, providing relevant parameters (e.g. latitude and longitude, elevation, water depth).*
Access and import/export	*Describe the efforts you have made to access habitats and to collect and import/export your samples in a responsible manner and in compliance with local, national and international laws, noting any permits that were obtained (give the name of the issuing authority, the date of issue, and any identifying information).*
Disturbance	*Describe any disturbance caused by the study and how it was minimized.*

Reporting for specific materials, systems and methods

We require information from authors about some types of materials, experimental systems and methods used in many studies. Here, indicate whether each material, system or method listed is relevant to your study. If you are not sure if a list item applies to your research, read the appropriate section before selecting a response.

Materials & experimental systems

n/a	Involved in the study
☐	☒ Antibodies
☒	☐ Eukaryotic cell lines
☒	☐ Palaeontology
☒	☐ Animals and other organisms
☒	☐ Human research participants
☒	☐ Clinical data

Methods

n/a	Involved in the study
☐	☒ ChIP-seq
☒	☐ Flow cytometry
☒	☐ MRI-based neuroimaging

Antibodies

Antibodies used	GST (B14; #sc-138; Santa Cruz), His (#R940-25; Life Technology), FLAG (#sc-807, Santa Cruz), H3 (#ab1791, Abcam), H3K9ac (#07-352, Millipore), H4K5ac (#07-327, Millipore), HTA9 (#AS10718, Agrisera), H2AZ.ac (#C15410173, Diagenode), H4K8ac (#07-328, Millipore), H4K12ac (#07-595, Millipore), H4K16ac (#07-329, Millipore)
Validation	All antibodies were validated by vendors.

Eukaryotic cell lines

Policy information about cell lines

Cell line source(s)	*State the source of each cell line used.*
Authentication	*Describe the authentication procedures for each cell line used OR declare that none of the cell lines used were authenticated.*
Mycoplasma contamination	*Confirm that all cell lines tested negative for mycoplasma contamination OR describe the results of the testing for mycoplasma contamination OR declare that the cell lines were not tested for mycoplasma contamination.*
Commonly misidentified lines (See ICLAC register)	*Name any commonly misidentified cell lines used in the study and provide a rationale for their use.*

Palaeontology

Specimen provenance	*Provide provenance information for specimens and describe permits that were obtained for the work (including the name of the issuing authority, the date of issue, and any identifying information).*
Specimen deposition	*Indicate where the specimens have been deposited to permit free access by other researchers.*
Dating methods	*If new dates are provided, describe how they were obtained (e.g. collection, storage, sample pretreatment and measurement), where they were obtained (i.e. lab name), the calibration program and the protocol for quality assurance OR state that no new dates are provided.*

☐ Tick this box to confirm that the raw and calibrated dates are available in the paper or in Supplementary Information.

Animals and other organisms

Policy information about studies involving animals; ARRIVE guidelines recommended for reporting animal research

Laboratory animals	*For laboratory animals, report species, strain, sex and age OR state that the study did not involve laboratory animals.*
Wild animals	*Provide details on animals observed in or captured in the field; report species, sex and age where possible. Describe how animals were caught and transported and what happened to captive animals after the study (if killed, explain why and describe method; if released, say where and when) OR state that the study did not involve wild animals.*
Field-collected samples	*For laboratory work with field-collected samples, describe all relevant parameters such as housing, maintenance, temperature, photoperiod and end-of-experiment protocol OR state that the study did not involve samples collected from the field.*
Ethics oversight	*Identify the organization(s) that approved or provided guidance on the study protocol, OR state that no ethical approval or guidance was required and explain why not.*

Note that full information on the approval of the study protocol must also be provided in the manuscript.

Human research participants

Policy information about studies involving human research participants

Population characteristics	*Describe the covariate-relevant population characteristics of the human research participants (e.g. age, gender, genotypic information, past and current diagnosis and treatment categories). If you filled out the behavioural & social sciences study design questions and have nothing to add here, write "See above."*
Recruitment	*Describe how participants were recruited. Outline any potential self-selection bias or other biases that may be present and how these are likely to impact results.*
Ethics oversight	*Identify the organization(s) that approved the study protocol.*

Note that full information on the approval of the study protocol must also be provided in the manuscript.

Clinical data

Policy information about clinical studies

All manuscripts should comply with the ICMJEguidelines for publication of clinical research and a completedCONSORT checklist must be included with all submissions.

Clinical trial registration	*Provide the trial registration number from ClinicalTrials.gov or an equivalent agency.*
Study protocol	*Note where the full trial protocol can be accessed OR if not available, explain why.*
Data collection	*Describe the settings and locales of data collection, noting the time periods of recruitment and data collection.*
Outcomes	*Describe how you pre-defined primary and secondary outcome measures and how you assessed these measures.*

ChIP-seq

Data deposition

- [x] Confirm that both raw and final processed data have been deposited in a public database such as GEO.
- [ ] Confirm that you have deposited or provided access to graph files (e.g. BED files) for the called peaks.

Data access links<br>*May remain private before publication.*	GEO link: https://www.ncbi.nlm.nih.gov/geo/query/acc.cgi?acc=GSE152940
Files in database submission	Raw FASTQ files, WIG files, tabular summary of read mapping statistics
Genome browser session<br>(e.g. UCSC)	Not applicable

Methodology

Replicates	ChIP-seq experiments were carried out in triplicates according to standard practice in the field.
Sequencing depth	Single-end data, 8-21 million reads per sample, read length: 50 bp. For Atepl1-2 H2A.Zac, samples with lower number of reads have been analyzed due to loss of this modification in the mutant background.
Antibodies	α-H3, Abcam ab1791, α-H4K5ac, Millipore 07327, α-H3K9ac Millipore 07352, α-HTA9 Agrisera AS10718 , α-H2A.Zac Diagenode C15410173
Peak calling parameters	Peak calling was not performed
Data quality	Peak calling was not performed
Software	For ChIP-seq adapters and poor-quality regions (mean Phred quality < 5) were removed from short reads using BBDuk2 program from BBTools suite (https://jgi.doe.gov/data-and-tools/bbtools). Filtered reads shorter than 35 bp were excluded from the analysis. Reads were then aligned to the A. thaliana (TAIR10) and mouse (GRCm38) genomes using BWA-MEM 0.7.17 aligner. Sambamba 0.7.0 was used to process the aligned data and remove duplicated reads. Mapping statistics, including the percentages of mapped reads and duplicates, were calculated separately for A. thaliana genome (ChIP-Seq data) and mouse genome ("spike-in" data) using BamTools 2.5.1. Normalized and smoothed coverage data used for ChIP-Seq plots was calculated using DANPOS 2.2.1.

Flow Cytometry

Plots

Confirm that:

- [ ] The axis labels state the marker and fluorochrome used (e.g. CD4-FITC).
- [ ] The axis scales are clearly visible. Include numbers along axes only for bottom left plot of group (a 'group' is an analysis of identical markers).
- [ ] All plots are contour plots with outliers or pseudocolor plots.
- [ ] A numerical value for number of cells or percentage (with statistics) is provided.

Methodology

Sample preparation	*Describe the sample preparation, detailing the biological source of the cells and any tissue processing steps used.*
Instrument	*Identify the instrument used for data collection, specifying make and model number.*
Software	*Describe the software used to collect and analyze the flow cytometry data. For custom code that has been deposited into a community repository, provide accession details.*
Cell population abundance	*Describe the abundance of the relevant cell populations within post-sort fractions, providing details on the purity of the samples and how it was determined.*
Gating strategy	*Describe the gating strategy used for all relevant experiments, specifying the preliminary FSC/SSC gates of the starting cell population, indicating where boundaries between "positive" and "negative" staining cell populations are defined.*

- [ ] Tick this box to confirm that a figure exemplifying the gating strategy is provided in the Supplementary Information.

Magnetic resonance imaging

Experimental design

Design type	*Indicate task or resting state; event-related or block design.*
Design specifications	*Specify the number of blocks, trials or experimental units per session and/or subject, and specify the length of each trial or block (if trials are blocked) and interval between trials.*
Behavioral performance measures	*State number and/or type of variables recorded (e.g. correct button press, response time) and what statistics were used to establish that the subjects were performing the task as expected (e.g. mean, range, and/or standard deviation across subjects).*

Acquisition

Imaging type(s)	*Specify: functional, structural, diffusion, perfusion.*
Field strength	*Specify in Tesla*
Sequence & imaging parameters	*Specify the pulse sequence type (gradient echo, spin echo, etc.), imaging type (EPI, spiral, etc.), field of view, matrix size, slice thickness, orientation and TE/TR/flip angle.*
Area of acquisition	*State whether a whole brain scan was used OR define the area of acquisition, describing how the region was determined.*

Diffusion MRI ☐ Used ☐ Not used

Preprocessing

Preprocessing software	*Provide detail on software version and revision number and on specific parameters (model/functions, brain extraction, segmentation, smoothing kernel size, etc.).*
Normalization	*If data were normalized/standardized, describe the approach(es): specify linear or non-linear and define image types used for transformation OR indicate that data were not normalized and explain rationale for lack of normalization.*
Normalization template	*Describe the template used for normalization/transformation, specifying subject space or group standardized space (e.g. original Talairach, MNI305, ICBM152) OR indicate that the data were not normalized.*
Noise and artifact removal	*Describe your procedure(s) for artifact and structured noise removal, specifying motion parameters, tissue signals and physiological signals (heart rate, respiration).*
Volume censoring	*Define your software and/or method and criteria for volume censoring, and state the extent of such censoring.*

Statistical modeling & inference

Model type and settings

Specify type (mass univariate, multivariate, RSA, predictive, etc.) and describe essential details of the model at the first and second levels (e.g. fixed, random or mixed effects; drift or auto-correlation).

Effect(s) tested

Define precise effect in terms of the task or stimulus conditions instead of psychological concepts and indicate whether ANOVA or factorial designs were used.

Specify type of analysis: ☐ Whole brain ☐ ROI-based ☐ Both

Statistic type for inference
(See Eklund et al. 2016)

Specify voxel-wise or cluster-wise and report all relevant parameters for cluster-wise methods.

Correction

Describe the type of correction and how it is obtained for multiple comparisons (e.g. FWE, FDR, permutation or Monte Carlo).

Models & analysis

n/a	Involved in the study
☐	☐ Functional and/or effective connectivity
☐	☐ Graph analysis
☐	☐ Multivariate modeling or predictive analysis

Functional and/or effective connectivity

Report the measures of dependence used and the model details (e.g. Pearson correlation, partial correlation, mutual information).

Graph analysis

Report the dependent variable and connectivity measure, specifying weighted graph or binarized graph, subject- or group-level, and the global and/or node summaries used (e.g. clustering coefficient, efficiency, etc.).

Multivariate modeling and predictive analysis

Specify independent variables, features extraction and dimension reduction, model, training and evaluation metrics.
